# Item Analysis of an Early Social Responsiveness Scale for Assessing Autism Risk

**DOI:** 10.3390/bs15050615

**Published:** 2025-05-01

**Authors:** Chloe Boynton, Opal Ousley, Reina S. Factor

**Affiliations:** 1 School of Medicine, Mercer University, Macon, GA 31207, USA; 2 Emory Autism Center, Decatur, GA 30033, USA; opal.y.ousley@emory.edu; 3 Semel Institute for Neuroscience and Human Behavior, University of California, Los Angeles, CA 90095, USA; rfactor@mednet.ucla.edu

**Keywords:** autism spectrum disorder, social responsiveness, screening

## Abstract

Early diagnosis of autism spectrum disorder (ASD) is vital for effective intervention and improves social and behavioral development. The previous literature has shown that the Early Social Responsiveness (ESR) assessment is effective at detecting ASD risk in individuals as early as 13 months of age (“parent study”). However, an item analysis that examines individual item scores has not been conducted to further elucidate the strength of this assessment. In this study, we analyzed an existing dataset (collected in the parent study) containing individual item responses from the ESR assessment of 120 children (*n* = 61 males and *n* = 59 females; age range = 15–24 months). Through item analysis, we determined which ESR items or item sets are best at differentiating ASD risk from non-ASD risk. Ease of social engagement (i.e., questions assessing the administrator’s perceived level of effort in engaging the child) was the most effective risk indicator, with the hat and tickle activities being least effective at indicating ASD risk. These results could contribute to optimizing the scale and facilitating its clinical adoption.

## 1. Introduction

Autism spectrum disorder (ASD) is a neurodevelopmental disorder characterized by impaired social interaction and communication, as well as repetitive behaviors, which includes sensory sensitivities (American Psychiatric Association, 2013; Grapel et al., 2015). Autistic children experience social differences early in development, which cause a gap between their social and behavioral development in comparison to typically developing children (Elder et al., 2017)^1^. The Center for Disease Control (CDC) reports that 1 in 36 children are diagnosed with ASD, with males being 3.8 times more likely to receive a diagnosis than females (Maenner et al., 2023; Shaw et al., 2020). However, this ratio might be lower, as there is emerging evidence suggesting underdiagnosis in autistic females (Lockwood et al., 2021). Clinician bias, complex phenotypic presentation, and an increased ability for females to mask symptoms, among many other factors, can contribute to missed diagnoses (Dell’Osso & Carpita, 2023). Additionally, research highlights phenotypic gender differences amongst autistic individuals. Primarily, autistic females show increased functional social behavior and less restricted and repetitive behaviors (RRBs) compared to male counterparts (Anagnostou et al., 2015). This highlights the importance of a balanced gender ratio among participants in future ASD studies.

Despite the high prevalence of autism, the average age of diagnosis is around 4 years, which may result in diminished social reciprocity between parent and child, a key component of family functioning and a core deficit of ASD, as well as later access to services (Maenner et al., 2023; Shaw et al., 2020). While it is suggested that ASD can be diagnosed most reliably at an age of 2 years, parents might observe behaviors indicative of ASD even earlier (Barton et al., 2012; Lord et al., 2006). This makes both clinical intervention and addressing parental concerns more difficult in early years. Thus, there is a need for earlier diagnosis, not only to provide earlier services but also for parental well-being (Factor et al., 2022; Watkins et al., 2017). Wan et al. (2013) found that qualities of caregiver–child interaction (e.g., more directive, lower dyadic mutuality, and intensity of engagement) in infants at risk for ASD were associated with ASD outcomes at 3 years of age. The Early Social Responsiveness (ESR) assessment has been shown to identify difficulties in early social interaction and differentiate children at high risk for ASD from peers (Factor et al., 2022). This interactive 3 min assessment measures specific social responsiveness behaviors (e.g., eye contact, smiling, pointing, turn taking, and ease of social engagement) across five activities observed in real time. The total score determines the ASD risk (Factor et al., 2022). Additionally, the assessment uses scripted instructions, providing an easier method for clinicians and pediatricians to adopt in clinic. Although initial results are promising, we do not know the effectiveness of each assessment item in determining ASD risk. The present study examines individual items to further explore the ESR assessment.

### 1.1. Early ASD Screeners

Early diagnosis of ASD, followed by effective intervention, is linked to improved development and notifies clinicians to examine co-existing medical conditions common in autistic individuals (Posar & Visconti, 2020). The American Academy of Pediatrics (AAP) suggests that children be screened for ASD as early as 18 and 24 months, but the stability of diagnoses at this age are not well supported (Guthrie et al., 2013). Early assessment tools utilize parental reports and interviews, as well as interactive assessments that examine social–behavioral skills. For example, the Communication and Symbolic Behavior Scales Developmental Profile (CSBS-DP) and the Social Communication Questionnaire (SCQ) are screening tools used to assess early social communication skills and can be used to identify at-risk children for ASD. However, there are concerns with their sensitivity in younger children and their ability to discriminate more ambiguous presentations (Saldaris et al., 2024; Moody et al., 2017). This emphasizes the importance of optimizing an early social communication screener like the ESR assessment in order to improve its efficacy and ease of clinical adoption.

Despite the variety of screeners available, the AAP report further indicates that there are no rapid infant screening tools (5 to 10 min) that allow for interactive assessment by the clinician. Barriers to adequate screening outlined by the report include (a) the absence of direct clinical assessments that can be administered by a general pediatrician or non-specialist; (b) the absence of a rapid assessment; and (c) the infrequent use of autism-specific standardized screening tools by pediatricians (Guthrie et al., 2013). Additionally, there is not currently a “gold standard” for early ASD risk assessment, and there is not a definitive biological ASD marker (Barton et al., 2012). While several genes increasing the susceptibility to ASD have been identified, a specific genetic contribution has not been defined. These genes are involved in biological pathways associated with other neurodevelopmental disorders; as such, the behavioral symptoms of ASD are shared among multiple etiologies (Molloy et al., 2011; Geschwind, 2011). Thus, these factors have resulted in the reliance on behavioral diagnostic measures (Geschwind, 2011). One of these is the Modified Checklist for Autism in Toddlers with Follow-up, revised (M-CHAT-23/F), which utilizes parents’ observations and can be used in a clinical setting. However, there is conflicting evidence on the accuracy, effectiveness, and reliability of the M-CHAT-23/F (discussed more below).

Common developmental behaviors that may indicate ASD risk include social communication skills, delayed speech, repetitive behavior (e.g., stimming), decreased eye contact, and little imitation or pretend play (Barbaro & Dissanayake, 2009; Posar & Visconti, 2020). A brief, comprehensive assessment that measures these domains would be a useful tool for identifying ASD risk early and allow greater access to early intervention opportunities. While the ESR assessment leverages the ability to measure the above-mentioned developmental domains in an efficient manner, barriers to clinical adoption prevent it from being widely administered.

### 1.2. Early Intervention and Access to Early Screening

The importance of early intervention programs for autistic individuals has continuously been highlighted (Watkins et al., 2017). Those who engage in intervention at younger ages tend to experience more social and behavioral gains compared to peers who access intervention later (Corsello, 2005). Intervention programs for ASD are numerous and vary in target skills, structure, and duration. Specific intervention targets may include social communication, language, play, joint attention (JA), inclusive learning environments, social skills, and behavioral challenges (Rogers & Dawson, 2020; LEAP; Boyd et al., 2014; Stahmer et al., 2020; Joint Attention Symbol Play Engagement Regulation; Shire et al., 2019; PCIT, Funderburk & Eyberg, 2011). Additionally, some interventions integrate a variety of caregivers such as teachers, clinicians, and parents as the main intervention administrators (Reichow et al., 2012). Despite different intervention targets and structures, earlier interventions are associated with better IQ outcomes, adaptive behavior, autism symptom severity, anxiety, and behavioral development (Dawson et al., 2010; Hyman et al., 2020; Reaven et al., 2012). Thus, the benefit of early interventions suggests the necessity to provide earlier screening.

Considering earlier screening and, therefore, greater access to early services, it is necessary to provide a fast, effective method for early pediatric assessment for atypical social responsiveness and consideration of ASD risk. Not all pediatricians regularly assess for ASD, although in 2013, the National Institute of Mental Health funded trials to develop innovations to improve early ASD screening and diagnosis (Fingert et al., 2019). However, a review of the implementation of these assessments determined multiple barriers preventing their adoption, such as inefficient systems of care, including provider attitudes (e.g., reluctance adopting new methods), and difficulty changing clinic processes (Fingert et al., 2019). One study determined continuous pediatric care and specialty referrals (e.g., behavioral therapists and pediatric neurologists) resulted in earlier diagnosis (Mandell et al., 2005). Thus, more routine clinical screening could positively impact early identification and treatment of autistic children.

While the barriers mentioned must be addressed, studies indicate that the implementation of assessment to detect ASD risk during well checkups is both possible and effective (Pierce et al., 2011). Parents’ concerns help detect 70–80% of children with disabilities, and 92% are able to complete questionnaires while in the exam room (Glascoe, 2000). Thus, parents’ surveillance of their children can provide healthcare professionals with vital information regarding the child’s level of ASD risk and allow experts to suggest interventions specific to the child’s needs. This work directly addresses the need for brief ASD assessments, outlined by the Centers for Disease Control and Prevention (CDC) and the AAP (Maenner et al., 2023; Myers & Johnson, 2007). Thus, the adoption of more routine screeners during checkups will allow for tailored interventions that can be administered sooner, providing greater potential for improved developmental outcomes.

### 1.3. Developmental ESR Behavior Related to ASD

Knowing the importance of these early social signs, early screening, and the necessity to create a brief, accurate, and accessible screener, ESR screening utilizes initial indicators of ASD to determine individual risk. This assessment measures social awareness, responsiveness, and engagement and captures developmental milestones typically present by the first year of life. These include an increase in smiling and vocalizations, attention to facial expression (autistic children tend to spend more time looking at objects than faces), and initiating JA, all of which indicate sharing experiences with others, a key component of social development (Factor et al., 2022). Systematic observation of an infant’s ESR may allow for the identification of subtle social delays, prior to the age when more obvious ASD symptoms emerge (Bryson et al., 2008). Young autistic children often exhibit fewer ESR behaviors and have difficulty in timing their social interactions (Vernon et al., 2013). Especially in activities involving JA, autistic infants struggle significantly more, resulting in poorer performance on the ESR assessment (Factor et al., 2022).

There is a well-established relationship between ASD and JA, with autistic individuals demonstrating decreases in response and the initiation of JA (Mundy & Crowson, 1997). Differences in JA, the ability to interact with another individual by capturing their focus, and directing the shared focus to an object, are among the earliest signs of autism. Thus, it has become the target for many intervention programs. Additionally, behaviors that involve JA can detect the developmental trajectory and severity of ASD (Charman, 2003). Due to the critical role JA plays in autism diagnosis, JA activities were included in the ESR assessment.

In addition to JA, other factors differentiate and define autism risk, which are also measured on the ESR assessment. Deficits in social and communication responsiveness are strongly associated with ASD, including reciprocal play, as autistic children struggle to imitate a play partner’s simple movements, initiate symbolic meaning, and act upon objects (Jaswal et al., 2020; Williams et al., 2004). Thus, reciprocal play with an object (i.e., rolling a ball back and forth) could be difficult for autistic children, a critical component of the ESR assessment. This is related to social engagement, the degree to which the child interacts in social activities with another individual. Social engagement is a key diagnostic feature of ASD, as well as a major component of the ESR assessment (Anagnostou et al., 2015). However, factors including age, language, and physical activity level might impact social engagement skills (Pan, 2009). Thus, the ESR assessment examines these domains and others for a holistic view of the child. Therefore, object-based activities and those requiring attention shifting, sustaining engagement, and expressing positive emotion within the ESR assessment could result in a higher reliability index (i.e., stronger correlation with the ESR total score) and validity scores (i.e., more predictive of an external criterion of ASD risk).

### 1.4. The Current Study

While a previous study found the total ESR score effectively distinguished ASD risk factors, the current study presents an item analysis of the ESR assessment using the same previously published data (Factor et al., 2022; “parent study”). Item analysis can improve and refine the assessment by investigating individual items, item sets, and the relationship between them (Desjardins & Bulut, 2020; McCowan & McCowan, 1999). Specifically, we calculated the item difficulty, item discrimination, item discrimination index, item reliability index, and item validity (described in more detail below).

The item analysis presented here tests which ESR items can best detect ASD risk and, therefore, suggests if a subset of items can be eliminated to further shorten the instrument. We predict the following outcomes: (1) JA items will be the most difficult assessment items; (2) every ESR item will discriminate between high and low ESR scorers; however, the JA items will have the strongest discriminatory ability; (3) each ESR item will discriminate between at-risk and not at-risk groups (i.e., above and below the median ESR total score); however, we anticipate JA items have a higher discrimination index; (4) all items will have high item reliability index (IRI), with object-based play tasks having higher IRI; and (5) all items will have high item validity (i.e., correlating with the external ASD risk criterion), with object-based play having the highest validity.

## 2. Materials and Methods

### 2.1. Participants

Statistical analyses were conducted on an existing dataset. Participants included a community subset of 120 children (*n* = 61 males and *n* = 59 females; age range = 15–24 months; mean age = 19.6 months; SD = 3.0). The sample included self-reported demographics, as shown in Table 1. Socioeconomic status (SES) is not explicitly reported. The highest level of education completed by the parent reflects access to resources that affect opportunity and social mobility, making it an effective surrogate measure (Khalatbari-Soltani et al., 2006).

Children completed assessments in English, including the ESR assessment, at the Child Study Lab at Georgia Institute of Technology. Pass/fail results on the M-CHAT-23/F revealed 11.7% (*n* = 14) of the participants were at risk for ASD (Table 1), and 40 (33.3%) children attended a follow-up appointment about 4 months after the initial visit (M = 4.6 months; SD = 1.8; range = 2–8 months). However, this study only looked at initial visit data.

### 2.2. Procedures

In the parent study, participants were given multiple assessments (45 min to complete). The present study only focused on the ESR assessment and pass/fail results on the M-CHAT-23/F. The recruitment of parent study participants included advertisements, flyers, and an online study portal, while more specific recruitment focused on children with known developmental disorders. All research staff who conducted the research visits held master’s or Ph.D. degrees and worked regularly within an ASD diagnostic clinic. Further, all instructions were standardized to decrease variability. Data were collected between March 2011 and June 2015. In accordance with university policies, the present study did not require IRB approval. As the study is a secondary data analysis and no new data were collected, informed consent was not required.

### 2.3. Measures

The following measures were completed in the parent study (Factor et al., 2022):

Modified Checklist for Autism in Toddlers with Follow-Up (M-CHAT-23/F; Robins et al., 2001): The M-CHAT-23/F is a parent-completed checklist that assesses the child’s current skills and behaviors. It consists of 23 yes/no items, is usually completed during a pediatric visit, and does not require the presence of a physician (Robins et al., 2001). A Low-Risk group is defined as having a score of 0 to 2 and does not require any action or follow-up interview (completed in the second study phase; not examined in the present study). A Moderate-Risk score requires a follow-up interview and a referral, with a score of 2 or more indicating ASD risk. A High-Risk score requires immediate referral (i.e., an interview is not required). Research has indicated that many M-CHAT-23/F questions successfully discriminate autistic children (18–24 months) from typically developing peers, and the questions best at accomplishing this capture JA skills (Robins et al., 2001; Wong et al., 2004). While reliability on the updated version of the M-CHAT-23 and follow-up has been indicated (Cronbach’s alpha = 0.79; Robins et al., 2014), there has been conflicting evidence on its effectiveness (discussed below).

Early Social Responsiveness (ESR) assessment (Factor et al., 2022): The ESR assessment takes 3 min, and both the child and parent are present. The child typically sits on the parent’s lap at a table, and the test is administered by trained clinicians or research assistants, which includes scripted language for item administration. Training consisted of live observation, practice administration with live feedback, and 80% or greater co-coding reliability. The ESR assessment includes five structured play activities with standardized verbal prompts and pauses to limit variability in administration or interpretation [e.g., saying “hello”, rolling a ball, looking at a book, a silly interaction (e.g., book on head), and tickling].

Twenty-two individual item scores are derived from the ESR assessment, including 17 behavioral codes and 5 summary codes. Behavioral codes indicate the presence/absence of pointing, eye contact, smiling, and turn taking. The absence of any behavior is recorded as a 1, and its presence is coded as a 0. The summary questions measure ease of social engagement on a 0–2 scale: very easy to engage (0), somewhat easy to engage (1), and hard to engage (2). However, a score of 1 is transformed into a 2 for scoring purposes. Therefore, the total score is the summation of the 17 behavior ratings, as well as the ease-of-social-engagement ratings (0–27). Higher scores indicate poorer performance, while lower scores indicate better performance.

More specifically, items 3, 8, 15, 18, and 22 in the ESR assessment measure ease of social engagement, items 4 and 9 measure JA skills, and items 5, 11, and 12 involve the child’s use of an object during play. All other items measure the presence or absence of social responsive behaviors during non-object play interactions (Appendix A). The instrument has strong internal consistency (α = 0.79) and test–retest reliability (*r* = 0.70).

### 2.4. Analytic Plan—Current Study

Preliminary analyses were conducted to provide sample demographics and summary statistics of all variables of interest. Data were analyzed to confirm that all assumptions of linear mixed-effects models were met.

Item analysis was conducted for each ESR question and totals using R. The following tests were run: item difficulty, item discrimination, item discrimination index, item reliability index, and the item validity. The meaning of each test is described below.

Item difficulty measures the proportion of participants who correctly answer each item (corresponding with an increase in total score), indicated by a *p*-value (Desjardins & Bulut, 2020). An item with a high *p*-value would be an easy question. This should not be confused with the *p*-value used for statistical significance. Given the reverse ESR coding, with 1 indicating the absence of a behavior and 0 meaning presence, item difficulty interpretation is flipped. Thus, the absence of behavior is equivalent to a “correct” answer, resulting in a higher total score, which suggests higher ASD risk, as it is harder for autistic children to perform.

Item discrimination refers to the ability of an item to distinguish between children who scored high on the assessment (i.e., high ASD risk) and those that scored low (i.e., low ASD risk). A point-biserial correlation between the individual item response and the total score was applied. Large, positive values indicate a strong correlation between answering an item correctly and doing well on the test (e.g., having a low total ESR score). Another way to calculate item discrimination (i.e., item discrimination index; IDI) is to split participants into two groups based on the total scores and correlate group membership to individual items (Desjardins & Bulut, 2020). We multiplied the odds ratio by the difference in the proportion of high and low performers who answered the question correctly. However, IDI is usually used for dichotomous answers; thus, we recoded individual item scores (e.g., for social engagement items, all scores of 2 were converted to a 1, and scores of 0 remained).

The Item reliability index (IRI) tests question reliability. High positive values indicate high reliability. The IRI can range from −0.5–0.5, although 0 or negative values are not often found. Results provided item statistics for the dataset, but only the r.drop was used, which is the comparison of the item against the scale if it were removed.

The Item validity index (IVI) is implemented when an external criterion is used. In this study, the external criterion is ASD risk, measured by pass/fail scores on the M-CHAT-23/F (Factor et al., 2022). Failing M-CHAT-23/F scores (high ASD risk) were coded 1, while children who passed (low ASD risk) were coded 0. The IVI scores range from −0.5–0.5, with absolute, larger values indicative of higher validity. Once this correlation was calculated, Fischer’s z transformation was used to convert the correlation coefficient to a normal distribution for ease of comparison.

## 3. Results

Data were determined to meet the assumptions of normality, linearity, and homoscedasticity. Descriptive statistics including means, standard deviations, and ranges were determined for each ESR assessment item (Table 2). Results are reported by item number (Appendix B).

### 3.1. Item Difficulty, Item Discrimination, and Item Discrimination Index

#### 3.1.1. Item Difficulty

Item difficulty is presented as a *p-value* but is not a measure of significance. The analysis showed that item 2 had the highest *p*-value (*p* = 0.825), with about 80% of children not smiling when greeted (Table 3). Items 10 and 14 (smiling [Item 10] and eye contact [Item 14]) also showed high *p*-values (*p* = 0.683 and *p* = 0.692), with about 68% and 69% of children either not smiling or unsuccessfully making eye contact after a pause. Items 16 and 19 had the lowest *p*-values (*p* = 0.008 and *p* = 0.067), with 99% of children making eye contact during the book-as-a-hat and tickling activities (Table 3). On JA measures, items 4 and 9, about 23% and 26% of participants did not demonstrate JA (Table 3).

#### 3.1.2. Item Discrimination

For items 8, 15, and 22, a large positive correlation was found (Table 3), suggesting that these items were best at discriminating high ESR scores from low ones. All three items measured ease of social engagement for the ball, book, and tickling activities.

#### 3.1.3. Item Discrimination Index

The *IDI* determined how the top and bottom scorers performed on each individual item (Table 3). Items 3, 8, 14, and 15 all had high IDI scores, ranging from 0.783 to 0.998 (3, 8, and 15—ease of social engagement). Items 16 and 19 (eye contact) had the lowest scores (Table 3 and Table 4).

### 3.2. Item Reliability and Item Validity

#### 3.2.1. Item Reliability

The IRI showed high values for items 8, 15, and 22 (social engagement), with no 0 values for any item (Table 3). The complete item statistics for item reliability analysis is shown in Table 4. The mean and standard deviations refer to each individual item and their individual scores.

#### 3.2.2. Item Validity

The IVI correlated ESR items with M-CHAT-23/F pass/fail scores. The correlations overall were low, with the highest value of 0.382 for item 4 (range = −0.031−0.382; Table 3). A negative correlation was found for item 20 (*r* = −0.031), which suggests that the item does not predict autism risk based on the M-CHAT-23/F. The correlation for the individual items to the total test score shows a high correlation for items 3, 8, 15, 21, and 22, ranging from 0.5 to 0.7 (Table 4, r.cor values). Specifically, items 3, 8, 15, and 22 are social engagement ratings.

## 4. Discussion

Early diagnosis and intervention are crucial to improving the developmental trajectory of autistic children or those who show early risk signs. However, many barriers prevent routine early screening. The ESR assessment was created to detect early delays in social responsive behaviors. While previous work indicates its strength as a screener, this study analyzed the individual items within the assessment to see if it could be shortened and improve clinical use (Factor et al., 2022).

The extant literature has indicated that JA skills are crucial to development and are delayed or deficient in autistic children, which can predict these children’s developmental trajectory (Charman, 2003; Factor et al., 2022; Mundy & Crowson, 1997). Thus, we predicted that ESR tasks involving JA would be the most accurate at differentiating children at high and low ASD risk. However, results suggest that ease-of-social-engagement measures are the best indicator of ASD risk. This could be due to its broader scope and earlier manifestation, while JA encompasses more specific behaviors (Dawson et al., 2012; Montagut-Asuncion et al., 2022). Item difficulty analysis demonstrates that eye contact in the hat and tickle activities was the easiest item, and the two JA measures were the 9th and 10th easiest out of 22 assessment items. Thus, difficulty engaging a child might emerge sooner and play a bigger role in early screening and intervention.

Item discrimination analysis showed that ease-of-social-engagement ratings in the ball, book, and tickling activities had the highest values, while eye contact in the hat activity had the lowest. This finding suggests that social engagement ability discriminates ASD risk status from a young age. Research has indicated that low levels of social engagement is associated with ASD severity, with high-risk children showing significantly less social engagement than their low-risk peers (Campbell et al., 2017). Therefore, these results again underscore the notion that social engagement is critical to determining ASD risk. IDI analyses further support this notion, as ease-of-social-engagement measures scored highest in the greet, ball, and book activities.

Ease-of-social-engagement items also had the highest reliability index scores in the ball, book, and tickle activities. Tasks involving objects ranked 13th, 14th, and 19th in reliability. The previous literature has shown that tasks involving the child’s use of an object poses a challenge for autistic children, as they spend more time looking at objects than faces (Pallett et al., 2014; Williams et al., 2004). However, this may highlight differences relating to social engagement. These findings are consistent with a survey suggesting that autistic children report struggling the most with social engagement, temper management, and social competence, highlighting the importance of social engagement in screeners (Knott et al., 2006).

Finally, JA in the ball activity had the highest validity value. However, the scores for the IVI, when using the M-CHAT-23/F as the external criterion, were low. Previous findings suggest that the M-CHAT-23/F indicates more positive results than other similar assessments and performs better when used in conjunction with other ASD screeners (Beuker et al., 2014). Additionally, the accuracy of the M-CHAT has been shown to be moderate for children aged 18–48 months, a portion of our sample (Charman et al., 2016). Thus, we believe that the low validity scores could be attributed to the fact that the M-CHAT-23/F might not be a sufficient measure of ASD risk. Further, when correlating ESR items to the total score, the validity of the items is much higher (Table 2). When this correlation is used, the ease-of-social-engagement scores in the ball, book, and tickle activities have the highest validity.

While parent questionnaires are a common method of early ASD screening, there exists conflicting evidence on the reliability of parents’ accounts. Recalled past events are often reported more recently than they happened, called forward telescoping (Ozonoff et al., 2018). While the ESR assessment utilizes real-time observations, there could be inaccuracies in parent observations reported on the M-CHAT-23/F. Additionally, an incorrectly reported onset could interfere with clinical interventions. Future studies should investigate observations on the ESR assessment and compare results to other provider administrations, as well as parent reports.

The results suggest that certain activities are more effective at detecting autism risk than others. There is evidence that the hat and tickle activities performed less effectively, with the hat activity often scoring near lowest. While the level of familiarity the child has with the activity/object could play a role, research has shown that with both novel and familiar objects, reciprocal play in children with ASD is the same, and thus, these tasks could be removed (Marsh et al., 2013). Another consideration is that physical activity has been found to correlate positively with social interaction and communication skills (Huang et al., 2020), and the ease-of-social-engagement rating, in the ball activity, was among the top scores across analyses. Ease-of-social-engagement rating items should be maintained in future iterations of the ESR assessment and other assessments, as these repeatedly suggested the strongest ability to detect ASD risk. However, the most informative type of activity used is not entirely clear. Further research could identify the skills required for each task and the best activity.

Social engagement is foundational to communication and vital components of successful development. Autistic children struggle with both understanding social interaction, as well as the performance of social interactive behaviors with others (Bauminger-Zviely et al., 2013). For social understanding, autistic children struggle with social norms, rules, and constructs, leading to deficits. The ESR assessment measures this by observing, in real time, interaction requiring social cues, object-based roleplay, and interpreting facial expressions. For the actual performance of social behaviors, joint attention, engagement, and play are crucial, all of which autistic children struggle to do and are measured in the assessment (Bauminger-Zviely et al., 2013). Interventions that target the understanding and the interaction of social engagement, as well as screeners that address both, have shown to be effective (Factor et al., 2022). In sum, all results indicate the significance of social engagement in determining ASD risk, even at an early age, which have implications in further crafting the most accurate ASD screener moving forward.

### 4.1. Limitations

We acknowledge that there are several limitations that should be addressed in future studies. First, the lower item validity scores when correlating individual items to the M-CHAT-23/F score should be considered. As previously mentioned, the M-CHAT-23/F has been found insufficient at detecting ASD risk independently (Beuker et al., 2014; Charman et al., 2016). While its sensitivity and specificity has been found to be better than other screening measures, a different validation measure should be considered, such as longitudinal follow-up or clinical diagnoses (Sunita & Bilszta, 2013). Additionally, the validity of the items could vary significantly with a different external criterion. Therefore, further analysis should be conducted before the consideration of clinical adoption.

Another limitation is the reliance on only one timepoint. Children attended a follow-up appointment about 4 months after the initial visit in the parent study, although this was not considered here (Factor et al., 2022). Autistic children can meet developmental milestones and later lose those skills, referred to as developmental regression (Al Backer, 2015). Some of the common skills lost include speech, nonverbal communication, social skills, and play skills (Rogers, 2004). While the ESR assessment does not require verbal speech, it does utilize play, nonverbal communication (i.e., eye contact), and social engagement. Thus, future studies should compare results from multiple administrations of the ESR test for a more reliable assessment of ASD risk. Additionally, the interpretation and execution of the scripted language in the assessment could vary between administrations, making analysis from multiple timepoints valuable.

Further, as the ESR test focuses heavily on difficulties in early social interaction, it might not be effective at differentiating ASD from other disorders with similar features. For example, Social Communication Disorder (SCD) is a neurodevelopmental disorder characterized by difficulties in language use for social aims (Topal et al., 2018). These disorders are distinguished by restrictive/repetitive behaviors seen in ASD, which could be captured during live observation during the ESR assessment (Gibson et al., 2013). Future research should analyze the ESR test’s ability to differentiate between these disorders.

### 4.2. Future Directions

As ASD screeners evolve and improve to become more accessible and accurate, it is necessary to consider which autistic behaviors are being measured. One study developed an Artificial Intelligence program that determined, from patterns of behavior between autistic children and their typically developing peers, that social skills, attention switching, and communication skills differed significantly between these groups (Shahamiri & Thabtah, 2020). These deficits in social behavior can persist throughout development, if not addressed by effective intervention (Vernon et al., 2013). By improving social engagement skills, autistic children are better able to communicate, form relationships, and exhibit emotional and social reciprocity (Koegel et al., 2012). This conclusion, highlighting the importance of social engagement skills, aligns with our findings that ease of social engagement was consistently the most effective risk indicator.

Future research should test various activities and measures included in the ESR assessment and compare the results to discover the best option. Additionally, these parameters should be evaluated in a gender-based analysis to explore how they perform in various phenotypic presentations. Importantly, ensuring that the objects required for the assessment are readily accessible in most households/offices and considering the length of the modified assessment will help increase the ease of administration and adoption in clinics. The consideration of shortening the assessment or making it available online could also add to the ease of implementation.

## 5. Conclusions

Autistic children experience social interaction and communication differences, which can impact their social and behavioral development. Diagnosis becomes most reliable at age 2, but concerning behaviors often arise before this age, making clinical intervention both necessary and less accessible. While the total ESR assessment score differentiates at-risk children from their peers, the effectiveness of individual items further highlighted the benefit of such an assessment, the need to specifically focus on social engagement, and the possibility of shortening the screener, making it easier to administer at routine appointments. A screener such as the ESR assessment could be more widely adopted and increase the ease of implementation for professionals and, thus, bolster families’ ability to take advantage of resources earlier and support their child throughout development.

## Figures and Tables

**Table 1:** Demographic information for community subset sample.

Variable		Percentage (*n*)
Child gender		
	Male	50.08 (161)
	Female	49.2 (52)
	Male/female ratio	1.03:1
MCHAT-23/ASD risk		11.7 (14)
Mother ethnicity		
	Asian	18.5 (5)
	Caucasian	70.40 (19)
Child ethnicity		
	African American	18.3 (22)
	Asian	11.66 (14)
	Caucasian	52.5 (63)
	Hispanic	0.83 (1)
	Multi-racial	10 (12)
	Other	1.66 (2)
	Unknown	5 (6)
Highest education level		
	Some high school	0.83 (1)
	High school diploma	5.8 (7)
	Some college	19.1 (23)
	College degree	33.3 (40)
	Some graduate school	8.3 (10)
	Graduate degree	25 (30)
	Other	1.7 (2)
	Did not report	5.8 (7)

**Table 2:** Descriptive statistics for the items in the ESR assessment.

	Mean	SD	Variance
1. Greeting: eye contact	0.167	0.374	0.140
2. Greeting: smile	0.825	0.382	0.146
3. Greeting: engagement	0.450	0.672	0.451
4. Ball: joint attention	0.225	0.419	0.176
5. Ball: roll	0.183	0.389	0.151
6. Ball: smile	0.100	0.301	0.091
7. Ball: pause	0.258	0.440	0.193
8. Ball: engagement	0.258	0.615	0.378
9. Book: joint attention	0.258	0.440	0.193
10. Book: smile1	0.567	0.498	0.218
11. Book: turn page	0.692	0.464	0.176
12. Book: point	0.575	0.770	0.246
13. Book: smile2	0.567	0.091	0.248
14. Book: pause	0.692	0.490	0.215
15. Book: engagement	0.608	0.467	0.593
16. Hat: eye contact	0.008	0.250	0.008
17. Hat: smile	0.608	0.382	0.24
18. Hat: engagement	0.158	0.464	0.218
19. Tickle: eye contact	0.067	0.25	0.063
20. Tickle: smile	0.175	0.382	0.146
21. Tickle: pause	0.308	0.464	0.215
22. Tickle: engagement	0.35	0.657	0.431

**Table 3:** Item analysis results for items in the ESR assessment.

	Item Difficulty	Item Discrimination	Item Discrimination Index	Item Reliability (r.drop)	Item Validity Index
1. Greeting: eye contact	0.167	0.509	0.429	0.446	0.261
2. Greeting: smile	**0.825**	0.339	0.292	0.265	0.031
3. Greeting: engagement	0.450	0.568	**0.783**	0.459	0.184
4. Ball: joint attention	0.225	0.495	0.467	0.424	0.381
5. Ball: roll	0.183	0.419	0.239	0.348	0.165
6. Ball: smile	0.100	0.425	0.258	0.371	0.052
7. Ball: eye contact after pause	0.258	0.436	0.332	0.356	0.331
8. Ball: engagement	0.258	**0.702**	**0.996**	**0.626**	0.372
9. Book: joint attention	0.258	0.416	0.328	0.334	0.142
10. Book: smile1	0.683	0.242	0.201	0.146	0.137
11. Book: turn page	0.225	0.432	0.361	0.355	0.179
12. Book: point	0.575	0.291	0.225	0.191	0.210
13. Book: smile2	0.567	0.447	0.555	0.357	0.162
14. Book: eye contact after pause	0.692	0.509	**0.896**	0.431	0.074
15. Book: engagement	0.608	**0.633**	**0.998**	**0.519**	0.153
16. Hat: eye contact	0.008	0.122	0.024	0.103	0.258
17. Hat: smile	0.608	0.393	0.437	0.300	0.187
18. Hat: engagement	0.158	0.498	0.352	0.418	0.214
19. Tickle: eye contact	0.067	0.320	0.067	0.271	0.111
20. Tickle: smile	0.175	0.453	0.479	0.386	−0.031
21. Tickle: eye contact after pause	0.308	0.534	0.522	0.458	0.210
22. Tickle: engagement	0.350	**0.605**	**0.781**	**0.506**	0.084

Note: Bolded values indicate high scores and are referred to in text.

**Table 4:** Item statistics of the item reliability analysis for the items in the ESR assessment.

	*n*	raw.r	std.r	r.cor	r.drop	Mean	SD
1. Greeting: eye contact	120	0.509	0.517	0.496	0.446	0.167	0.374
2. Greeting: smile	120	0.339	0.354	0.301	0.265	0.825	0.382
3. Greeting: engagement	120	0.568	0.539	**0.529**	0.459	0.450	0.672
4. Ball: joint attention	120	0.495	0.527	0.498	0.424	0.225	0.419
5. Ball: roll	120	0.419	0.404	0.380	0.348	0.183	0.389
6. Ball: smile	120	0.425	0.441	0.403	0.371	0.100	0.301
7. Ball: pause	120	0.436	0.427	0.384	0.356	0.258	0.440
8. Ball: engagement	120	0.702	0.671	**0.686**	**0.626**	0.258	0.615
9. Book: joint attention	120	0.416	0.446	0.414	0.334	0.258	0.440
10. Book: smile1	120	0.242	0.241	0.180	0.146	0.683	0.467
11. Book: turn page	120	0.432	0.441	0.403	0.355	0.225	0.419
12. Book: point	120	0.291	0.268	0.208	0.191	0.575	0.495
13. Book: smile2	120	0.447	0.451	0.414	0.357	0.567	0.497
14. Book: pause	120	0.509	0.492	0.462	0.431	0.692	0.464
15. Book: engagement	120	0.633	0.591	**0.585**	**0.519**	0.608	0.770
16. Hat: eye contact	120	0.122	0.196	0.141	0.103	0.008	0.091
17. Hat: smile	120	0.393	0.389	0.333	0.299	0.608	0.490
18. Hat: engagement	120	0.498	0.506	0.481	0.418	0.158	0.467
19. Tickle: eye contact	120	0.320	0.356	0.318	0.271	0.066	0.250
20. Tickle: smile	120	0.453	0.453	0.427	0.386	0.175	0.382
21. Tickle: pause	120	0.534	0.559	0.536	0.458	0.308	0.464
22. Tickle: engagement	120	0.605	0.589	**0.588**	**0.506**	0.350	0.657

Note: “raw.r” = correlation of item with total score; “std.r” = correlation of item with total score if items are standardized; “r.cor” = item whole correlation corrected for item overlap and scale reliability; “r.drop” = item whole correlation against scale without item (Revelle, 2022). Bolded values indicate high scores and are referred to in text.

## Data Availability

Data are stored and available upon request from opal.ousley@emory.edu.

## Note

1 Self-advocates from the autism community (Bury et al., 2020) and current research have indicated a preference for identify-first language (e.g., autistic children). Therefore, this language will be utilized throughout the manuscript.

## Appendix A

**Table A1:** The Early Social Responsiveness Scale.

Instructions for the interactive assessment of early social responsiveness			
Step 1: Present each task in a standardized way. For each task, follow the instructions in the shaded boxes. For Task 2, use a ball about 3 inches across. For Task 3, use a board book between 2 and 4 inches. Step 2: Rate the target behaviors. Answer the questions about the child’s behavior, as you complete each task. 0 = Yes, the child showed the behavior. 1 = No, the child did not show the behavior. Step 3: Rate ease of engagement for each task. Complete the engagement ratings, as you complete each task. 0 = Yes, very easy to engage—this rating indicates that the child is attuned to the examiner’s actions, is readily available for interaction, and attends to the examiner with anticipation and expectancy, requiring minimal effort from the examiner 1 = Somewhat easy to engage 2 = Hard to engage Step 4: Determine the Total Score. In the far-right column, circle the appropriate score or converted score that corresponds to each rating. Sum all scores in the far-right column to determine a Total Score.			
TASK 1 Smiling and saying “hello”	Instructions: When you are ready to start, smile and say in a playful tone, “Hi (insert child’s name).” PAUSE for 2 s and say, “Are you ready to play with some new toys?” Lean in and keep smiling for 2 s.		
	Did the child look you in the eyes?	0 = Yes 1 = No	0 1
	2. Did the child smile?	0 = Yes 1 = No	0 1
	3. Engagement rating: Overall, during Task 1, was the child easy to engage, taking little to no effort from you?	0 = Yes, very easy to engage; child attends to the examiner with *anticipation* and *expectancy* 1 = Somewhat easy to engage (→ converted to a score of 2) 2 = Hard to engage	0 2
TASK 2 Ball play	Instructions: Hold the ball to the right, about 12 inches from your head, at your eye level. Say “Look at my ball.” Watch to see if child looks at the ball then back to your eyes.		
	4. Did the child look at the ball and then back at your eyes?	0 = Yes 1 = No	0 1
	Instructions: Say “Let’s play ball. Ready, set, GO!” See if child will roll or throw the ball back to you, then repeat at least 2 times but not more than 4.		
	5. Did the child roll or throw the ball back to you one or more times?	0 = Yes 1 = No	0 1
	6. Did the child smile?	0 = Yes 1 = No	0 1
	Instructions: On the 3rd roll say, “Ready, set……” PAUSE for 5 s “GO!”		
	7. Did the child look at your eyes after the PAUSE?	0 = Yes 1 = No	0 1
	8. Engagement rating: Overall, during Task 2, was the child easy to engage, taking little to no effort from you?	0 = Yes, very easy to engage; child attends to the examiner with *anticipation* and *expectancy* 1 = Somewhat easy to engage (→ converted to a score of 2) 2 = Hard to engage	0 2
TASK 3 Book	Instructions: Hold the book to the right, about 12 inches from your head, at your eye level. Say, “Look at my book.”		
	9. Did the child look at the book and then back at your eyes?	0 = Yes 1 = No	0 1
	10. Did the child smile?	0 = Yes 1 = No	0 1
	Instructions: Present the book, within 6 inches in front of child, as you read the 1st page. Modeling turning the page, read the 2nd page, then say “Let’s see what’s next” (wait for the child to turn the page). If the child does not turn the page, turn the page and say, “Where is the (insert name of picture in the book)?” only once. Then say, “Can you turn the page?” at least once but not more than twice. Then continue turning the pages at least 3 times consecutively (see below for PAUSE instructions).		
	11. “Did the child turn one or more pages?”	0 = Yes 1 = No	0 1
	12. Did the child point to or tap on a picture in the book?	0 = Yes 1 = No	0 1
	13. Did the child smile?	0 = Yes 1 = No	0 1
	Instructions: After 3 consecutive page turns, say “Let’s see what’s next……” PAUSE for 5 s as you hold the page with your thumb, preventing the child from turning the page…… then turn the page.		
	14. Did the child look at your eyes after the PAUSE?	0 = Yes 1 = No	0 1
	15. Engagement rating: Overall, during Task 3, was the child easy to engage, taking little to no effort from you?	0 = Yes, very easy to engage; child attends to the examiner with *anticipation* and *expectancy* 1 = Somewhat easy to engage (→ converted to a score of 2) 2 = Hard to engage	0 2
TASK 4 Putting book on your head as a hat	Instructions: As you playfully put the book on your head, *gasp* while smiling. Then say, “Where’s the book?” Wait 2 s, then say, “It’s on my head, it’s a hat!”		
	16. Did the child look you in the eyes?	0 = Yes 1 = No	0 1
	17. Did the child smile?	0 = Yes 1 = No	0 1
	18. Engagement rating: Overall, during Task 4, was the child easy to engage, taking little to no effort from you?	0 = Yes, very easy to engage; child attends to the examiner with *anticipation* and *expectancy* 1 = Somewhat easy to engage (→ converted to a score of 2) 2 = Hard to engage	0 2
TASK 5 Smiling and tickling	Instructions: Hold your hands up in front of you, wiggle your fingers, and say “I’m gonna tickle you.” Wait 2 s, then say “I’m gonna get you, I’m gonna get you, I’m gonna get you” while *slowly* leaning in toward the child. *Gently* tickle the child on the belly or arms, saying “tickle, tickle, tickle” Repeat this sequence for a 2nd time, starting with I’m gonna get you, … etc”.		
	19. Did the child look you in the eyes?	0 = Yes 1 = No	0 1
	20. Did the child smile?	0 = Yes 1 = No	0 1
	Instructions: On the 3rd time begin again, as above, and say, “I’m gonna get you…..” then PAUSE for 5 s before saying, “I’m gonna get you, I’m gonna get you” and then gently tickling, etc.		
	21. Did the child look at your eyes after the PAUSE?	0 = Yes 1 = No	0 1
	22. Engagement rating: Overall, during Task 5, was the child easy to engage, taking little to no effort from you?	0 = Yes, very easy to engage; child attends to the examiner with *anticipation* and *expectancy* 1 = Somewhat easy to engage (→ converted to a score of 2) 2 = Hard to engage	0 2
	TOTAL SCORE: Higher scores indicate poorer early social responsiveness skills *(range = 0–27).*		

Note: originally from (Factor et al., 2022).

## Appendix B

### R Codes

Item Difficulty

“item_diff <- colMeans(ESR_Item_Scores, na.rm = TRUE)”.

round(item_diff, 3

*Item Discrimination*

total_score <- rowSums (ESR_Item_Scores, na.rm = TRUE

item_discr <- cor(ESR_Item_Scores, total_score)

Item Discrimination Index

high_performers <- subset(ESR_Item_Scores, total_score > median(total_score)) low_performers <- subset(ESR_Item_Scores, total_score <= median(total_score))

p_high <- mean(high_performers$item_response)

p_low <- mean(low_performers$item_response)

model <- glm(item_response ~ performance_score, data = your_data, family = binomial())

coefficients <- coef(model)

odds_ratio <- exp(coefficients [2])

log_odds_high <- coefficients[1] + coefficients[2] *median(performance_score) log_odds_low <- coefficients[1]

idi <- odds_ratio * (p_high - p_low)

Item Reliability Index

reliability <- alpha(ESR_Item_Scores)

reliability.index <- item.stats$alpha.drop

Item Validity

item.stats <- alpha(ESR_Test_Items)$item.stats

validity.index <- item.stats$r.cor

itemval <- cor(ESR_Item_Scores, ESR_Item_Scores_With_MCHAT$MCHAT valindex <- fischerz(itemval)

Descriptive Statistics

Sapply(ESR_Item_Scores, var)

Sapply(ESR_Item_Scores, sd)

Sapply(ESR_Item_Scores, mean)

Summary(ESR_item_Scores)

Note: The “psych” package in R was downloaded and used for some analyses.
